# Legacies of domestication, trade and herder mobility shape extant male zebu cattle diversity in South Asia and Africa

**DOI:** 10.1038/s41598-018-36444-7

**Published:** 2018-12-21

**Authors:** Lucía Pérez-Pardal, Alejandro Sánchez-Gracia, Isabel Álvarez, Amadou Traoré, J. Bento S. Ferraz, Iván Fernández, Vânia Costa, Shanyuan Chen, Miika Tapio, Rodolfo J. C. Cantet, Ajita Patel, Richard H. Meadow, Fiona B. Marshall, Albano Beja-Pereira, Félix Goyache

**Affiliations:** 1 Servicio Regional de Investigación y Desarrollo Agroalimentario, E-33394 Gijón, Spain; 2 CIBIO - InBIO, Universidade do Porto, 4485-661 Vairão, Portugal; 3 Department de Genètica, Microbiologia i Estadística and Institut de Recerca de la Biodiversitat, Universitat de Barcelona, 08028 Barcelona, Spain; 4 Institut de l’Environnement et des Recherches Agricoles (INERA), Ouagadougou, 04 BP 8645 Burkina Faso; 5 Department de Ciencias Básicas, Faculdade de Zootécnia e Engenharia de Alimentos, Universidade de São Paulo, Pirassununga, 13635-900 SP Brazil; 6 School of Life Sciences, Yunnan University, Kunming, China; 7 Natural Resources Institute Finland (Luke), Helsinki, FI-00790 Finland; 8 Department de Producción Animal, Universidad de Buenos Aires-INPA-CONICET, Buenos Aires, C1427CWO Argentina; 9 Peabody Museum, Harvard University, Cambridge, MA 02138 USA; 10 Department of Anthropology and Peabody Museum, Harvard University, Cambridge, MA 02138 USA; 11 Department of Anthropology, Washington University in St. Louis, St. Louis, MO 63130 USA

## Abstract

All tropically adapted humped cattle (*Bos indicus* or “zebu”), descend from a domestication process that took place >8,000 years ago in South Asia. Here we present an intercontinental survey of Y-chromosome diversity and a comprehensive reconstruction of male-lineage zebu cattle history and diversity patterns. Phylogenetic analysis revealed that all the zebu Y-chromosome haplotypes in our dataset group within three different lineages: Y3_A_, the most predominant and cosmopolitan lineage; Y3_B_, only observed in West Africa; and Y3_C_, predominant in South and Northeast India. The divergence times estimated for these three Zebu-specific lineages predate domestication. Coalescent demographic models support either *de novo* domestication of genetically divergent paternal lineages or more complex process including gene flow between wild and domestic animals. Our data suggest export of varied zebu lineages from domestication centres through time. The almost exclusive presence of Y3_A_ haplotypes in East Africa is consistent with recent cattle restocking in this area. The cryptic presence of Y3_B_ haplotypes in West Africa, found nowhere else, suggests that these haplotypes might represent the oldest zebu lineage introduced to Africa ca. 3,000 B.P. and subsequently replaced in most of the world. The informative ability of Interspersed Multilocus Microsatellites and Y-specific microsatellites to identify genetic structuring in cattle populations is confirmed.

## Introduction

The last two decades of genetic studies on the origin of livestock species have unveiled a remarkable set of new questions regarding the origin and spread of domesticated animals. Research on single versus multiple domestications, interbreeding of wild and domestic animals, and cultural and faunal exchanges is transforming current perspectives on domestication trajectories and biodiversity^1–3^. Genetic studies of cattle domestication have pointed out that modern cattle (*Bos taurus* and *B. indicus*) resulted from at least two genetically distinct auroch populations (*B. primigenius* and *B. nomadicus*)^4,5^. The domestication of *B. taurus* in the Near East and expansion of taurine cattle into Europe has received particular attention^6–8^. However, studies of the domestication and spread of the zebu cattle lineage, *B. indicus*, are relatively scant.

Archaeological findings suggest that zebu cattle were domesticated 8,000–9,000 years ago (B.P.) and dispersed throughout northwestern South Asia by 6,000 years before present^9,10^. South India may have been an additional centre for cattle domestication^11,12^, as may have been Gujarat in western India^9,11–13^. Several areas of domestication or wild capture are consistent with genetic studies, which have identified two major mtDNA haplogroups (or lineages) in zebu cattle: I1 and I2^4^. An extensive survey of modern cattle suggests that the maternal zebu cattle lineage I1 likely originated from the domestication of local wild cattle (*Bos namadicus*) in northwestern South Asia^4^. Animals of this lineage may have spread through South Asia (≈5,500–4,000 years B.P.) and beyond the Subcontinent eastwards to Southeast Asia and southern China (by ca. 2,500 B.P.)^4^. After this initial spread, additional genetic diversity was recruited to domestic herds from South Asian wild cattle populations carrying the haplogroup I2.

In comparison with mtDNA^4^ and genome-wide^14^ information studies conducted on *B. indicus* analyses of Y-chromosome diversity are rare. Seminal studies using Single Nucleotide Polymorphisms (SNPs) identified a single zebu Y-chromosome lineage (Y3)^15^. Admixture between *B. taurus* and *B. indicus* in Africa and differences in diversity parameters assessed using Y-chromosome markers^16–18^ have contributed to understand the complexity of domestication processes, the early spread of male zebu cattle in South Asia and the introduction of zebu to Africa through Indian Ocean trade.

Humped zebu-like cattle were introduced to Egypt from the Levant in small numbers and depicted in tombs and temples 3,400–3,000 B.P.^19^ but present African zebu cattle populations are the result of multiple introductions. Archaeology suggests that most animals came through eastern rather than northern Africa. Pre-Aksumites, Aksumites and their trading partners in Yemen and the Red Sea brought zebu cattle to the Horn of Africa 2,000–1,600 B.P.^20,21^ following well known sea trade routes from South Asia to the Gulf of Oman^22,23^. Humped cattle followed Sahelian routes, appearing in West Africa by 1,000 B.P.^24^. The rinderpest panzootics of 1889–1896 are estimated to have annihilated up to 80% of herds in many regions or over 5.2 million African taurine cattle^25^ and resulted in zebu being massively reintroduced along the eastern coastline of Africa, largely replacing African taurine bulls.

Genetic data have demonstrated that African cattle mainly carry the maternal T1 taurine mtDNA lineage^26^. However, the African zebu and sanga populations (crosses between African taurine and zebu cattle) mainly carry Y-chromosomes of zebu origin^17^. The absence of zebu mitochondria in African cattle implies that introgression of the zebu lineage into Africa was mostly male mediated. A number of microsatellite-based studies have also shown that zebu introgression into Africa declines from East to West^26^. Moreover, a study using Y-chromosome markers, found that the zebu-specific Y-chromosome sub-lineage Y3_B_^27^ was absent from the extant zebu population from the Indian subcontinent. Very recently, the X-degenerate region within the male-specific part of the bovine Y-Chromosome was resequenced^28^ allowing to identify two sub-haplogroups within the *B. indicus* Y3 chromosome: the Y3_a_ sub-haplogroup identified in Chinese cattle and the Y3_b_ sub-haplogroup mainly carried out by Indian zebu sires. However, African zebu was not included in that analysis^28^. Here, we report on the genetic analysis of a large number of West African and South Asian zebu Y-chromosomes to shed light on the origin and spread of zebu cattle within south Asia and from south Asia to Africa.

## Methods

### Sampling

Samples from 248 *B. indicus* males belonging to 22 cattle populations from Asia and Africa were analysed (Supplementary Table S1). The Asian dataset totalled 100 made up of three Indian native populations (from northeastern, Central and southern Indian areas; totalling 26 samples) plus unrelated samples from major zebu breeds kept in Argentina and Brazil that descend from pure zebu sires from India: Brahman (6), Gir (10), Guzerat (5), Indubrazil (2), Nelore (46) and Tabapuã (5). When necessary, samples from Brahman, Indubrasil and Tabapuã breeds were pooled into a single population due to their historical admixed origin (which included the use of Guzerat, Nelore and Gir individuals). We consider this pooled Indian population to originate from a geographical location that averages those of parental populations. We also analysed samples from 5 sires (obtained in 3 different countries) from Central Asia and 8 sires from Yemen. The African dataset totalled 135 samples, from East Africa (3 populations and 38 samples) and West Africa (6 populations, including Central African M’Bororo cattle, and 97 samples).

### Genotyping

The two Y-specific Interspersed Multilocus Microsatellites (IMMs; UMN2405 and UMN2303) and the six Y-chromosome specific microsatellites (INRA189, UMN0103, UMN0307, BM861 and BYM1)^16^, with two loci typed for microsatellite UMN0103^29^ were genotyped following a protocol described previously^27^. We have adopted the terminology recommended by the Human Y-Chromosome Consortium^30^.

IMM bands and microsatellite alleles were combined into haplotypes. Observed haplotypes were analysed as follows: (a) Analysis of correspondence was performed using the “Proc Corresp” of the SAS/STAT package (SAS Institute Inc., Cary, USA); (b) applying the Bayesian procedures implemented in the program MrBayes^31,32^. Two MCMC runs starting from different random trees were completed. Each run consisted of 20 million replications and four chains. All sample points prior to reaching convergence were discarded as burn-in samples. The remaining samples were used to generate a majority rule consensus tree, where the percentage of samples recovering any particular clade represented the clades posterior probability^32^; and (c) a MJ network connecting different haplotypes was constructed using the program Network 4.5.2^33^ (available at http://www.fluxus-engineering.com/). To avoid reticulation, a reduced median algorithm^34^ was used to generate an.rmf file to which the median joining network method^33^ was applied to this file. Following the recommendations of^35^, the “frequency >1” option was applied to discard singly occurring Y-types. The same weights were assigned to each polymorphism.

### Phylogenetic and population genetic analyses

An unbiased estimate of haplotype diversity, *h*, and its variance, *V*(*h*), were calculated according to Nei^36^ (formulas 8.5 and 8.13 therein). The standard error of *h*, *SE*(*h*), was calculated by taking the $\sqrt{V(h)}$. Within population gene diversity adjusted for sampling size was also computed following Nei^36^. The between-populations genetic identity matrix was computed as the average across loci of the term to $\sum _{ij}\,{x}_{ij}{y}_{ij}$ ^36^,where *x*_*ij*_ and *y*_*ij*_ are the frequencies of the *i*^*th*^ allele at the *j*^*th*^ locus within the populations *x* and *y*, using the program MolKin^37^. The information provided by the between-population genetic identity was summarized, from the complementary of this matrix, using a PCA. The PC scores and the canonical dimensions computed for each analysed population were used to construct interpolation maps drawn using the Spatial Analyst Extension of ArcView, available at http://www.esri.com/software/arcview/. The Inverse Distance Weighted (IDW) option with a power of two was selected for the interpolation of the surface. IDW assumes that each input point has a local influence that decreases with distance. The sampling area of each population was used as geographic coordinates and interpolation surfaces were divided into eight equal classes.

### Demographic scenarios

**Figure 1:**
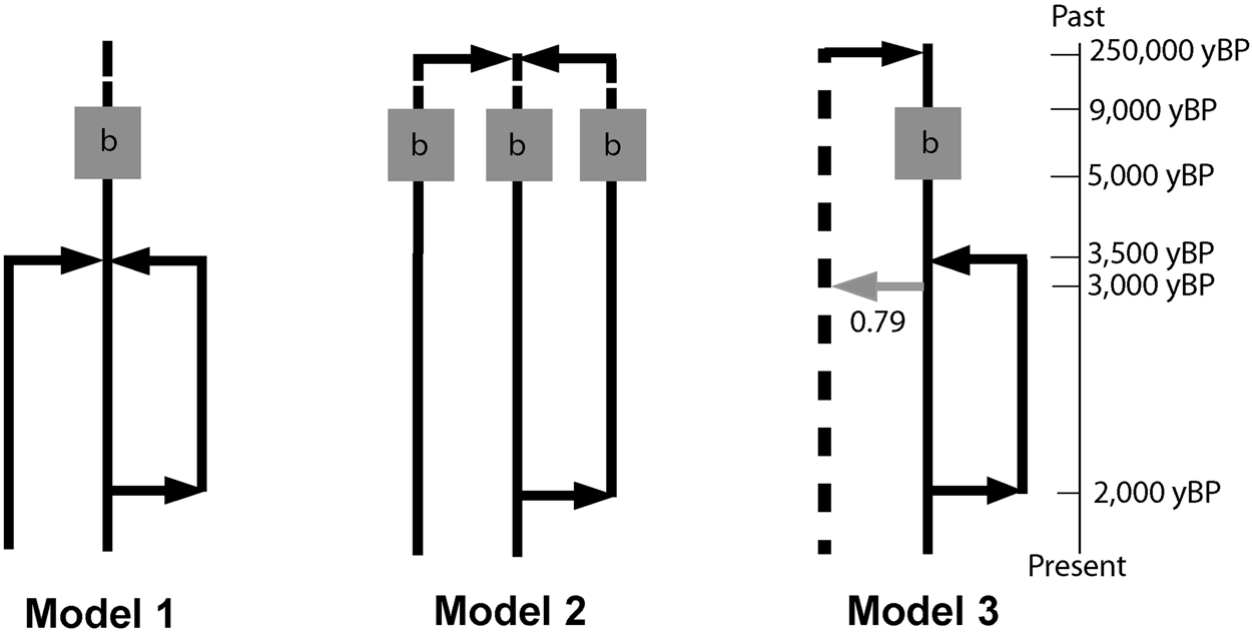
Schematic view of the demographic models compared in the ABC model choice. Model 1, a single domestication followed by the diversification in three descendant lineages. Model 2, three independent domestications or early recruitment from three differentiated ancestral populations. Model 3, a wild oxen admixture (grey arrow) between a single domesticated lineage and an ancestral differentiated population (with a proportion of admixture input into the domesticated lineage of 0.79^50^. In all cases, the complete admixture of two of the descendant lineages occurred 2,000 B.P. The “b” within the grey square illustrates the bottleneck associated with domestication. Arrows represent the direction of the events backwards in time (coalescent simulations). The last admixture at time 0 (present) was included in the coalescent simulations to account for the artificial mixture caused by the unique sample. Details of the parameters used for simulations are provided in the Materials and Methods section.

Three historical scenarios for domestication and the spread of zebu cattle were modelled (Fig. 1) using the coalescent sampler implemented in SIMCOAL 2.1.2^38^. We refer only to the initial capture of males as domestication (≈9,000 years ago^1,2^) and refer to later instances of capture and recruitment of wild bulls to herds as introgression event or gene flow. Model 1 assumed an ancestral Y-chromosome population undergoing a bottleneck associated with a single domestication and subsequent population split at the first introduction of zebu into Africa (ca. 3,500 B.P.^19,20^) into three different populations, two of them being merged at 2,000 B.P.; Model 2 assumed three different ancestral Y-chromosome domesticated populations evolving separately during 250,000 years^39^, with two of them being merged at 2,000 B.P.; Model 3 assumed two ancestral Y-chromosome domesticated populations one of them splitting into two subpopulations at 3,500 B.P. (which, in turn, merge at 2,000 B.P. like in the other two Models) and received migrants in an introgression event at 3,000 B.P. from the other population of the ancestral lineage. The scenarios modelled represent simple (Model 1) or complex (Models 2 and 3) domestication processes that originated the present admixed zebu Y-chromosome population. Model 3 represents the more complex, and sustained in time, domestication scenario including a dominant ancestral Y-chromosome population which acts as a genetic source of the others. The merging of two of the ancestral populations at 2,000 B.P. represents a secondary, less intense, recruitment of wild oxen, probably out of the Indus Valley, suggested by mtDNA structure^4^. Current effective population sizes (10^3^–10^5^ individuals), IMMs mutation rate (ranging from 2 × 10^−5^ to 2 × 10^−4^ mutation/generation) and the size of the initial domesticated populations (1–200 individuals) were modelled assuming uniform distributed priors. Divergence times were measured in years and transformed to generations for simulations using a generation time of 4.84 years^40^. For Y-chromosome microsatellites, rapidly mutating markers mutation rate^41^ (0.008 mutations/generation) was used under a strict stepwise mutation model. The two IMMs, which are considered slow mutation dominant markers^27^, were simulated as independent markers. Both the IMMs and the six different microsatellite loci typed were simulated using SIMCOAL for 180 individuals. This latter figure coincides with the number of available non-American zebu Y-chromosomes plus those haplotypes that were only observed in American zebu.

The posterior probability of each proposed scenario was then calculated using the software ABCtoolbox^42^ with 10^6^ simulation replicates generated under each scenario and using four summary statistics, the standard deviation over loci of the number of alleles (*K*_*sd*_), the mean heterozygosity over loci ($\bar{H}$) and standard deviation over loci of the heterozygosity (H) and the mean total heterozygosity ($\overline{Ht}$) calculated with the software alrsumstat^43^. Half a percent (0.5%) of the simulations matching closest the empirical data based on distances between observed and simulated statistics was retained for the estimation of the marginal densities under the General Linear Model (GLM)^44^ for each evolutionary scenario. These were also used for the assessment of the posterior odds (Bayes factors)^45^ for each model given the observed data. The ABC model choice was validated using simulated replicates under the three models as pseudo-observed datasets to estimate the power to distinguish between models, the percentage of model misclassification and the posterior probabilities supporting wrong choices^43^.

### Divergence times estimation

Divergence times were estimated via the *ρ* statistic (i.e. the average number of mutations from derived haplotypes) to a haplotype designated as ancestral for the haplogroup^18,46^. The average distance to the node of interest (*ρ* was transformed to absolute time estimates by multiplication (*ρ* × years per 1 mutation). As a phylogeny-based statistic, ρ offers the advantage of being unbiased by demographic processes. The sampling error of *ρ* was approximated as $\sqrt{\frac{{\boldsymbol{\rho }}}{n}}$, where n denotes the sample size. Due to the fact that our dataset included IMMs, that are likely to be dominant slowly-mutating markers^27^ and rapid mutating Y-chromosome microsatellites, divergence times were computed assuming the average mutation rate of 0.0008/generation reported in^39^ for intermediate- rate mutating Y-chromosome markers.

### Additional resequencing

The very recent identification of two sub-haplogroups (Y3_a_ and Y3_b_) within the zebu Y3 Chromosomes by Chen *et al*.^28^ made necessary carrying out additional analyses to ascertain their consistency with the variability identified in the current analysis. A 443pb fragment (from position 3631054 to 3631601; reference assembly Btau_5.0.1 GCF_000003205.7) of the X-degenerate region within the male-specific part of the bovine Y-Chromosome was resequenced for diagnostic purposes. The amplified fragment included three mutations (g.3631254a > g, g. 3631400t > c and g. 3631401t > g) separating the Chen *et al*.’s^28^ *B. indicus* Y-Chromosome sub-haplogroups Y3_a_ and Y3_b_. A total of 51 sequences were obtained. A complete description of the laboratory methods used is in Supplementary Table S2.

## Results

Sixteen of the bands (seven for UMN2405 and nine for UMN2303) described for the IMMs used in Pérez-Pardal *et al*.^27^ were polymorphic (in terms of presence/absence) in zebu cattle. The number of alleles per microsatellite varied from one (BM861) to four (smaller loci of the marker UMN0103, BYM1 and INRA189), revealing a total of 19 alleles across the six loci here considered (Supplementary Table S3).

We identified 47 different haplotype combinations resulting from the genotypes of two IMMs and six microsatellites markers. Of these, 29 are unique. The three most frequent haplotypes (H19, 24 samples; H20, 37; and H21, 55) were identified in 18 different populations. However, these three haplotypes were neither found in non-Indian Asian samples nor in sires from northeastern India. Haplotype H24 was identified in 23 samples from the six West African populations analyzed (Supplementary Table S3). Within-population haplotypic diversity ranged from 0.089 ± 0.015 in Nelore cattle to 0.800 ± 0.213 in Ethiopian Raya-Azebo and Nigerian Goudali cattle (Supplementary Table S1). Within-population gene diversity varied from 0.014 in Nelore cattle to 0.181 in Malian Bororo cattle. Regarding geographic areas, (not considering the strongly bottlenecked American zebu samples) the highest haplotypic and gene diversity values were found in non-Indian Asian samples (0.583 ± 0.101 and 0.202, respectively) or Indian (0.500 ± 0.008 and 0.209, respectively) sires. Overall gene diversity and haplotype diversity were 0.152 and 0.190, respectively (Supplementary Table S1).

**Figure 2:**
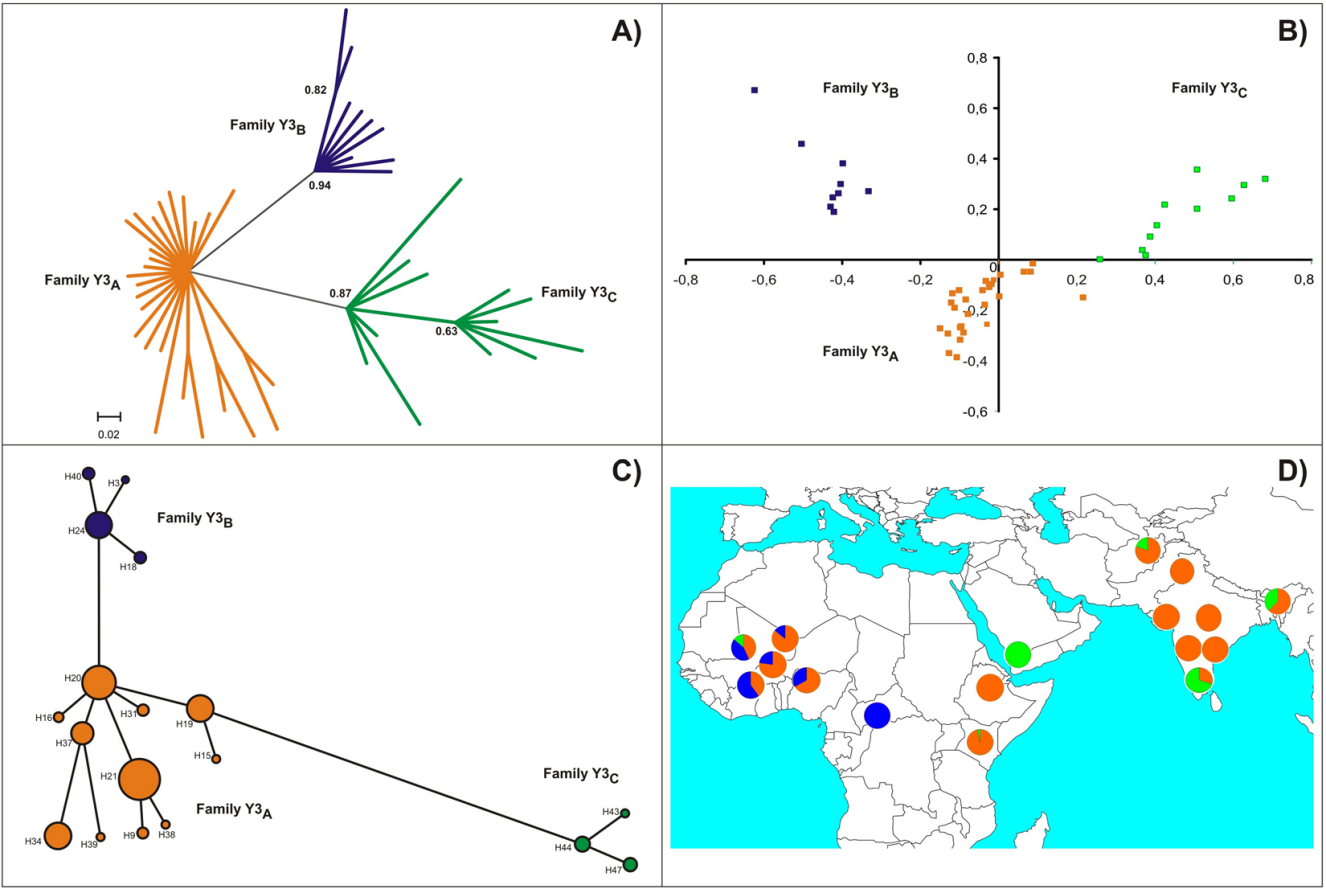
Graphical representation of genetic diversity and phylogeny of the zebu specific Y-chromosome lineages. Plot (**A**) shows the phylogenetic tree constructed from the 146 identified haplotypes using the Bayesian procedures implemented in the program MrBayes 3.1.Plot (**B**) shows the two dimensions calculated on the polymorphism of the 47 Y-chromosome haplotypes identified via correspondence analysis; and Plot (**C**) shows a network tree constructed using the program Network 4.5. The three identified haplotypic families (Y3_A_, Y3_B_ and Y3_C_) are, respectively, in orange, blue and green. Plot (**D**) shows a geographic map and the frequency of the haplotypic families identified. Here, size of the circles does not correspond with sample size.

All the Bayesian phylogeny, median-joining (MJ) network and the correspondence analyses concur in the identification of three Y3 haplotypic families (Fig. 2). The main one, Y3_A_, included 26 haplotypes and 186 samples (55% and 75% of the total haplotypes and samples, respectively). The Y3_B_ included 12 (25%) haplotypes and 24 (10%) samples, while the Y3_C_ included 9 (20%) haplotypes and 38 (15%) samples (Supplementary Table S1). When compared with haplotypic family Y3_A_, the Y3_B_ is mainly defined by the presence of three bands (126, 127 and 128) on the IMM UMN2303, while family Y3_C_ is defined by the absence of the allele 149 on microsatellite UMN0307 and the predominant presence of band 124 on IMM UMN2303 (Supplementary Table S3). The Bayesian analysis provided a statistically significant confidence (0.94) for the separation between haplotypic families Y3_A_ and Y3_B_ while that between families Y3_A_ and Y3_C_ was slightly lower (0.87). Correspondence analysis separated the haplotypic families Y3_B_ and Y3_C_ on Dimension 1 (X-axis) while the main haplotypic family (Y3_A_) was differentiated on Dimension 2 (Y-axis). The network obtained using the whole dataset was highly reticulated (Supplementary Fig. S1). However, after removal of singly occurring haplotypes^35^ the network obtained was highly consistent with the other analyses (Fig. 2C) identifying H20, H24 and H44 as the central haplotypes within families Y3_A_, Y3_B_ and Y3_C_, respectively. Observed haplotype families also showed differential geographic frequencies (Fig. 2D). The Y3_A_ was predominant in India and was present in all populations sampled except for Yemen and The Central African Republic. Haplotypic family Y3_B_ was only present in West Africa while Y3_C_ was mainly present in South India and Yemen and only has traces in Northeast India, Central Asia and East Africa.

Between-population genetic relationships were assessed via Principal Components Analysis (PCA; Supplementary Table S4) and graphically visualized on synthetic maps (Supplementary Fig. S2). The first three principal components (PC) with eigenvalue >1, explain 65.5, 16.4 and 7.4% of the total variability. The first PC differentiates the genetic variability of most northern Indian subcontinent and Central Asian samples (mainly carrying Y3_A_ haplotypes) from those of Southern India and Yemen (where family Y3_C_ haplotypes are present). The second PC contrasts the genetic variability of the Yemen, and southern and northeastern India populations with those samples from West Africa in which haplotypes belonging to family Y3_B_ are frequent (Supplementary Fig. S2). Consistency of this geographically-related analysis was evaluated by a second PCA from which zebu individuals of Argentinean and Brazilian origin were excluded. This latter analysis allowed identification of two PCs with eigenvalue >1, explaining 64.7 and 17.1% of the total variability (not shown).

Three historical scenarios (Fig. 1) for the domestication and spread of zebu cattle were modelled using the obtained data. Two of the three models compared under the ABC model choice (Model 1 and Model 2) had high relative posterior probabilities (PP), with Bayes factors (BF) of 8.9 and 6.4 with Model 3. Therefore, Model 3 was clearly disposable. Although Model 2 was the most probable model, its probability is only slightly higher than that of Model 1 (BF = 1.4), which is insufficient statistical evidence to draw clear conclusions about a possible separate domestication of different zebu Y-chromosome populations. Nevertheless, our cross-validation study of the statistical properties of the ABC model choice indicated that this slight difference in probabilities might be enough to seriously consider Model 2 as the best candidate. This claim is supported on the fact that, when comparing the three models tested based on pseudo-observed data sets (PODS; Supplementary Fig. S3), the estimated posterior probability favouring Model 2 given real data (PP = 0.548) was never reached in false positives (i.e., when the true model that generated the data was Model 1).

Moreover, Model 2 was additionally run considering the admixture of the three descendant populations at 2,000 B.P. This scenario was less supported by the observed data (results not shown), indicating that two of the descendants of these domesticated populations would share recent history but a third population would remain isolated (or partially isolated) until the present. This fact leaded us to retain the hypothesis underlying Model 2 as more plausible.

Resequencing (Supplementary Table S2) informed that sequences assigned to the haplotypic family Y3_A_ and to the “African” haplotypic family Y3_B_ identified here can be included into the Chen *et al*.’s^28^ *B. indicus* Y-Chromosome sub-haplogroup Y3_b_, identified in zebu sires of Indian origin, while the sequences assigned to the current the haplotypic family Y3_C_ carried mutations g.3631254a > g, g. 3631400t > c and g. 3631401t > g and, therefore, are consistent with the definition of the Chen *et al*.’s^28^ *B. indicus* Y-Chromosome sub-haplogroup Y3_a_, mainly identified in Chinese zebu sires.

## Discussion

The diversity observed in the zebu cattle lineage was lower than that for taurine cattle at the same Y-chromosome loci^16,27^. In keeping with the early history of movement of the lineage, *B. taurus* Y-chromosomes belong to two different haplogroups (Y1 and Y2)^15^. All zebu cattle Y-chromosome haplotypes fall within a distinct Y3 haplogroup. More specifically, zebu cattle Y-chromosomes can be classified into three different Y3 haplotypic families (Fig. 2). However, the low genetic variability found is consistent with a short divergence between these haplotypic families (Table 1) indicating a short divergence between the wild populations involved in the domestication process. The ABC model choice used to compare the support of the data to three possible domestication histories points towards a scenario of separated domestication for each the three Y-chromosome families identified or multiple early and regionally diverse events of gene flow as likely, with no simple discrimination among them possible. With the revelation of how common gene flow is among domestic animals and their wild relatives today researchers are leaning towards requiring a high bar for identification of multiple domestication^1,2,47^. In the light of the validation study carried out, however, the results of the ABC model choice must be interpreted as additional evidence to be considered together with other information, such as projection of zebu cattle Y-chromosome genetic variation on geographical maps, to clarify to the history of the species.

**Table 1:** Divergence times between ancestral haplotypes estimated using *ρ* estimates.

Reference haplotype	H20-H24	H20-H44	H24-H44
Haplotypic family	Y3_A_-Y3_B_	Y3_A_-Y3_C_	Y3_B_-Y3_C_
*ρ* (SD)	1.15 (0.66)	1.59 (0.16)	3.03 (0.23)
Years (SD)	6,957 (4,017)	9,625 (962)	18,351 (1,411)

Results were computed assuming an intermediate mutation rate of 0.0008 per generation.

The comparison of the findings of the current study with recently reported by Chen *et al*.^28^ give insights of general interest as well:

- The consistency of our majority haplotypic families Y3_A_ and Y3_B_ with the Chen *et al*.’s^28^ *B. indicus* Y-Chromosome sub-haplogroup Y3_b_ is in agreement with the fact that haplotypic families Y3_A_ and Y3_B_ had the lower divergence times (6,957 ± 4,017 years; Table 1). Therefore, it can be hypothesized that sires carrying Y3_A_ and Y3_B_ haplotypes could belong to the same or closely related domesticated populations. Furthermore, the Indian origin of our “African” Y3_B_ haplotypic family is confirmed.
- The consistency of our South and Northeast India haplotypic family Y3_C_ with the Chen *et al*.’s^28^ *B. indicus* Y-Chromosome sub-haplogroup Y3_a_ clarifies that this “Chinese” sub-habplogroup is likely to have an Indian subcontinent origin. Therefore, the significance of the Indian subcontinent in the domestication of *B. indicus*^4^ is confirmed again.

### History of zebu cattle

Despite the fact that the Indian subcontinent was the centre of domestication of zebu cattle^9,10^, the haplotypic family Y3_B_ was only observed in African zebu. These data most likely reflect the effect of waves of introduction of zebu to Africa. The total absence of Y3_B_ haplotypes in South Asia suggests interbreeding between local African and domestic Asian cattle, the traces of which were swamped by subsequent population shifts in South Asia, after the linage was introduced to Africa.

Currently available archaeological data indicates that humped cattle were not introduced to Africa earlier than ≈3,500 B.P.^19,20^ and that humped cattle may have reached these areas from abroad in small numbers^19,21,24,48^. The range of breeding choices made by the varied owners of zebu cattle are unknown^49^, but only zebu sires have provided a genetic signal traceable to the present^50^.

Considering that all three zebu-specific Y3 sub-lineages discovered in this research originated in the domestication centre, the exclusivity of the Y3_B_ haplotypes in West Africa zebu sires can only be explained by the history of zebu movements and population demographic events. It is likely that this Y3_B_ haplotypic family derived from an ancient South Asian process of domestication and was later replaced by herder’s recruitment of new male lineages from wild stock into the zebu gene pool.

### The origin of West African zebu

The exclusivity of the Y3_B_ haplotypic family in West African zebu cattle is significant as a reservoir of male biodiversity^27^. Given the significance of males for breeding programs we think that these populations should be further explored and seriously considered in any cattle biodiversity conservation program. Within the Indian subcontinent, the presence of the Y3_C_ family in southern and southeastern India (Fig. 1) is also notable, particularly, when considering that the zebu mtDNA lineage (I2), is projected to date around ≈3,500 B.P.^4^. An early expansion of cattle-oriented Neolithic cultures carrying recent domesticated zebu cattle from the Indus Valley eastward into the Indo-Gangetic plains, and southward into the southern tip of the Indian subcontinent^4,9,11^, may have resulted in introgression of local wild stock into domesticated herds. In fact, population size estimates for *B. indicus* can only be explained if one assumes a substantial and posterior admixture event between the bottlenecked early domestic zebu population and wild animals^39^. Our estimates can support the posterior recruitment of I2 females as well as introgression of Y3_C_ sires into early zebu herds. In the light of archaeological and ethnographic data it is likely that a range of practices in different regions of the continent resulted in wild-domestic gene flow, including extensive herding, herders intentional turning out domestic cows to breed with wild bulls, and wild capture to maintain hardiness and herd sizes^2,9–11,51^.

The elevated frequency of the haplotypic family Y3_A_ in our African dataset may represent recent zebu introductions over the past two centuries, which have increased the frequency of Y3_A_ sires in East Africa. The restocking of African herds with animals carrying Y3_A_ haplotypes suggests that this lineage was already the most frequent in South Asia. Samples are smaller from Yemen, but given the pivotal role of the Gulf of Oman trade routes from India in ancient plant exchanges and traditional maritime coastal trading routes to the Horn of Africa, the absence of the Y3_A_ lineage raises the question of whether nineteenth century introductions of East African zebu followed southerly Indian Ocean routes^22^ (Y3_C_; Supplementary Table S1).

The observed geographic patterns have led us to speculate that the first zebu males transported to Africa were mostly from Y3_B_ and Y3_C_ lineages and that recent introductions brought the Y3_A_ lineage to Africa. As the eastern coast of Africa was more exposed to the influence of zebu introductions, the older Y3_B_ and Y3_C_ were replaced by the Y3_A_ lineage, especially in the last 200 years. The Atlantic coast of Africa, on the other hand, was less exposed to the influence of these maritime routes, and the distance from eastern Africa was a factor in the persistence of the older Y3_B_ male zebu lineage in western Africa cattle today. These findings of our research demonstrate how a livestock species such as cattle, has expanded through time in ways in which one wave of expansion might erase the former one, with persistence of unexpected biodiversity linked to ancient histories of trade and exchange.

## Ethics Statement

Blood and hair root samples were collected by veterinary practitioners with the permission and in presence of the owners. For this reason, permission from the Ethics Committee for Health Research in Burkina Faso (Joint Order 2004-147/MS/MESSE of May 11, 2004) was not required. In all instances, veterinarians followed standard procedures and relevant national guidelines to ensure appropriate animal care. Semen doses were routinely collected by different companies not directly related to our research project.

## Electronic supplementary material

Supplementary Information

Supplementary Dataset 1

## Data Availability

All genotypes, haplotypes and sequences obtained are provided as Supplementary Table S3 (xlsx file).
