# Supplementary material for: Legacies of domestication, trade and herder mobility shape extant male zebu cattle diversity in South Asia and Africa
Source: Sci Rep. 2018 Dec 21;8:18027. doi: 10.1038/s41598-018-36444-7 (PMC6303292; doi:10.1038/s41598-018-36444-7)

Supplementary Information for

Legacies of domestication, trade and herder mobility shape extant male zebu cattle diversity in South Asia and Africa

Lucía Pérez-Pardal, Alejandro Sánchez-Gracia, Amadou Traoré, J. Bento S. Ferraz, Isabel Álvarez, Iván Fernández, Vânia Costa, Shanyuan Chen, Miika Tapio, Rodolfo J. C. Cantet, Ajita Patel, Richard H. Meadow, Fiona B. Marshall, Albano Beja-Pereira, Félix Goyache[1]

[1]To whom correspondence and requests should be addressed (e-mail: fgoyache@serida.org)

This PDF file includes:

Supplementary Table S1: Description of sampling and within population diversity.

Supplementary Table S2: Laboratory methods and polymorphism identified on a fragment of the X-degenerate region within the male-specific part of the bovine Y-Chromosome.

Supplementary Table S4: Between-populations genetic identity matrix and eigenvectors corresponding to each of the three factors computed via Principal Component Analysis.

Supplementary Figure S1: Network tree illustrating the genetic variability found with the haplotypes constructed.

Supplementary Figure S2: Synthetic maps illustrating geographic variation of the first and second principal components identified using PCA.

Supplementary Figure S3: Results of the ABC model choice misclassification.

Supplementary Table S3, containing all the genotypes and haplotypes obtained, is provided in a separated Excel file.

Supplementary Table 1. Distribution of the 248 zebu male individuals analyzed per sampling area and population (defined by country of origin or breed). The number of samples per haplotype family ($Y3_A$, $Y3_B$ and $Y3_C$) and population is also detailed. Additionally, each population is assigned to a given geographical area: a) Non-Indian Asia (including Yemen and Central Asian samples; 13); b) India (100 samples); c) East Africa (including Ethiopian and Kenian samples; 38); and d) West Africa (including Burkina Faso, Mali, Nigeria and Central African Republic samples; 53). The following parameters are also given for populations with sample size equal to or greater than 5: unbiased gene diversity (H) and haplotype diversity corrected for sample size (*h*) and its standard error ($SE_{(h)}$). Samples obtained in Central Asia and Ethiopia were pooled.

Sampling Area	Population	Sample size	$Y3_A$	$Y3_B$	$Y3_C$	H	n	*h*	$SE_{(h)}$
Arabian Peninsula	Yemen	8			8	0.024	3	0.429	0.070
Central Asia		5	4		1	0.171	4	1.000	0.305
	Afghanistan[1]	2	1		1		2		
	Kazakhstan[1]	1	1				1		
	Turkmenistan[1]	2	2				1		
India	Brahman[2]	6	6			0.000	1	0.200	0.010
	Gir	10	10			0.046	3	0.333	0.037
	Guzerat	5	5			0.086	4	1.000	0.305
	NorthEast India	8	5		3	0.213	7	1.000	0.259
	Central India	8	8			0.080	5	0.714	0.164
	Southern India	10	3		7	0.164	4	0.444	0.070
	Indubrasil[2]	2	2				1		
	Nelore	46	46			0.014	4	0.089	0.015
	Tabapuá[2]	5	5			0.019	2	0.500	0.105
Kenya	Improved Boran	29	28		1	0.067	10	0.357	0.029
Ethiopia		9	9			0.066	6	0.750	0.169
	Danakil[3]	3	3				2		
	Raya_Azebo[3]	6	6			0.083	4	0.800	0.213
Burkina Faso									
	Lobi	15	6	9		0.111	6	0.429	0.056
	Zebu_Peul[4]	35	27	8		0.099	8	0.235	0.007
Central African Republic	Mbororo[4]	6		6		0.063	4	0.800	0.213

Mali									
	Bororo[4]	28	12	12	4	0.181	12	0.444	0.046
	Azaouak	7	6	1		0.041	2	0.333	0.039
Nigeria	Goudali	6	4	2		0.108	4	0.800	0.213
Totals		248	186	38	24	0.152	47	0.190	0.000
Geographical Area									
Non-Indian Asia		13	4		9	0.202	7	0.583	0.101
India and American zebu		100	90		10	0.119	31	0.313	0.012
India (local samples)		26	16		10	0.209	13	0.500	0.008
East Africa		38	37		1	0.072	16	0.432	0.038
West Africa		97	53	38	4	0.134	36	0.375	0.018

[1]Samples from Afghanistan, Kazakhstan and Turkmenistan were pooled into the 'Central Asia' population.

[2]For geographical analyses, the Brahman, Indubrasil and Tabapuá populations were pooled and considered to be originated in a geographical location 'averaging' that corresponding to their parental populations (Guzerat, Nelore and Gir populations).

[3]Samples from the Danakil and Raya Azebo populations were pooled into the 'Ethiopia' population

[4]The Malian Bororo, Burkinabé Zebu Peul and Central African M'Bororo populations belong to the West African Zebu population, kept in the Sahel area by the Fulani (also known as Peul or Bororo) ethnical group. The Fulani are nomadic herdsmen, spread from Sudan in the East to Senegal and Mauritania in the West, representing a major force for genetic exchange all over central and West Africa.

Supplementary Table S2. Laboratory methods and polymorphism identified on a fragment of the X-degenerate region within the male-specific part of the bovine Y-Chromosome.

A primer set (Primer Forward: ACTCCAGACTCTCCCTGGAC; Primer Reverse: GCATTTACACTTTAGCGGGTGA) was manually designed to amplify a fragment of the bovine Y-Chromosome sequence from position 3631054 to 3631601 according to the reference assembly Btau_5.0.1 (GCF_000003205.7). The size of the amplicon obtained was 443 bp. The amplified fragment included three mutations separating the Chen *et al.*'s (2018, *Nat. Commun.* **9**, 2337) *Bos indicus* Y-Chromosome sub-haplogroups $Y3_a$ and $Y3_b$ (defined by mutations g.3631254a>g, g. 3631400t>c and g. 3631401t>g). Chen *et al.* (2018) identified the sub-haplogroup $Y3_a$ on Chinese cattle and sub-haplotype $Y3_b$ on three *B. indicus* breeds of Indian origin.

Laboratory methods were as follows: The polymerase chain reaction (PCR) protocol consisted of a 20-µl reaction mix containing approximately 50 ng of total DNA, 0.5 µM of both forward and reverse primer, DNA AmpliTools Master Mix (Biotools, Madrid, Spain). Amplification was carried out for 35 cycles at an annealing temperature of 60 °C in a GenAmp 9700 thermocycler (Applied Biosystems, Madrid, Spain). PCR products were purified using ExoSAPIT (USB, Cleveland, OH) following the manufacturer's recommendations. PCR products were sequenced using an ABI 3130 automated DNA sequencer and the ABI Prism Big Dye Terminator 3.1 Sequencing Kit (Applied Biosystems, Madrid, Spain).

Table summarises the polymorphism identified on a total of 51 sequences previously assigned to the following the $Y3_A$, $Y3_B$ and $Y3_C$ haplotypic families (23, 23 and 5, respectively). The 23 sequences previously assigned to the haplotypic family $Y3_A$ belong to Brahman (2), Lobi (2), Malian Bororo (4), Zebu Azaouak (3), Zebu Peul (5), Goudali (1), Raya Azebo (1), Afghan zebu (1) and Boran (2) sires; the 23 sequences previously assigned to the haplotypic family $Y3_B$ belong to Lobi (5), Malian Bororo (10), Zebu Azaouak (1), Zebu Peul (5), Goudali (1) and Mbororo (1) sires; finally, the 5 sequences previously assigned to the haplotypic family $Y3_C$ belong to Malian Bororo (4) and Afghan zebu (1) sires.

The whole correspondences found are in the sheet "Correspondence" of the Supplementary Table S3 given as an Excel file. The analysis of the polymorphism informed that both the sequences assigned to the majority haplotypic family $Y3_A$ and to the "African" haplotypic family $Y3_B$ identified in the current analysis can be considered as belonging to the Chen *et al.*'s (2018) *B. indicus* Y-Chromosome sub-haplogroup $Y3_a$, identified in zebu sires of Indian origin, while the sequences assigned to the current the haplotypic family $Y3_C$ carried mutations g.3631254a>g, g. 3631400t>c and g. 3631401t>g and, therefore, are consistent with the Chen *et al.*'s (2018) *B. indicus* Y-Chromosome sub-haplogroup $Y3_b$, mainly identified in Chinese zebu sires.

	Haplotypic family*	Position (bp)			Chen et al.'s (2018) sub-haplogroup**
		3631254	3631400	3631401	
Reference allele		A	T	T	
Alternative allele		G	C	G	
chinese$Y3_b$**		A/A	T/T	T/T	
chinese$Y3_a$**		G/G	C/C	G/G	
23 sequences*	$Y3_A$	G/G	C/C	G/G	$Y3_b$
23 sequences*	$Y3_B$	G/G	C/C	G/G	$Y3_b$
5 sequences*	$Y3_C$	A/A	T/T	T/T	$Y3_a$

Supplementary Table S4. Between-populations genetic identity matrix computed using two IMMs and six Y-microsatellite loci typed on 248 zebu sire DNA samples. The 'Central Asia' population was constructed pooling samples from Afghanistan, Kazakhstan and Turkmenistan. The 'Ethiopia' population was formed pooling the Danakil and Raya Azebo samples. The 'Pooled India' population was constructed using the samples obtained from the Brahman, Indubrasil and Tabapuá breeds. Those populations that do not carry haplotypes belonging to family $Y3_B$ usually had genetic identity higher than 0.9. Eigenvectors corresponding to each of the three factors computed via Principal Component Analysis are given for each population. Breeds in which the main haplotypic family $Y3_A$ is predominant have high and positive eigenvectors for Factor 1. Those breeds in which the $Y3_C$ haplotypes are predominant have negative eigenvectors for Factor 1. Breeds with high and positive eigenvectors for Factor 2 are those with predominant composition of $Y3_B$ haplotypes while those with negative eigenvectors for Factor 2 are those in which $Y3_C$ haplotypes are present. Factor 3 does not have clear population-based patterns of variation.

Population	1	2	3	4	5	6	7	8	9	10	11	12	13	14	15	16	17	Factor 1	Factor 2	Factor 3
1. Improved Boran																		0.788	0.553	0.065
2. Bororo	0.849																	0.215	0.794	-0.260
3. Central Asia	0.857	0.772																0.936	-0.041	-0.156
4. Ethiopia	0.919	0.825	0.877															0.904	0.317	-0.071
5. Gir	0.916	0.839	0.845	0.893														0.762	0.550	0.187
6. Goudali	0.899	0.859	0.808	0.863	0.885													0.437	0.825	-0.162
7. Guzerat	0.907	0.819	0.870	0.911	0.927	0.863												0.905	0.356	-0.020
8. NorthEast India	0.825	0.760	0.770	0.804	0.812	0.794	0.789											0.163	-0.025	0.774
9. Pooled India	0.902	0.803	0.904	0.929	0.874	0.852	0.925	0.771										0.936	0.227	-0.213
10. Central India	0.906	0.817	0.875	0.912	0.919	0.863	0.930	0.787	0.935									0.917	0.332	-0.058
11. Southern India	0.740	0.693	0.701	0.714	0.750	0.705	0.712	0.775	0.666	0.706								-0.322	-0.493	0.689
12. Lobi	0.856	0.850	0.764	0.829	0.852	0.883	0.828	0.749	0.804	0.825	0.675							0.186	0.874	-0.336
13. M'bororo	0.792	0.830	0.706	0.765	0.775	0.857	0.759	0.677	0.750	0.758	0.588	0.887						0.032	0.806	-0.563
14. Nelore	0.911	0.837	0.831	0.882	0.969	0.882	0.926	0.810	0.854	0.915	0.755	0.853	0.772					0.713	0.581	0.230
15. Yemen	0.702	0.662	0.667	0.683	0.705	0.667	0.667	0.789	0.623	0.661	0.868	0.641	0.548	0.707				-0.420	-0.550	0.666
16.Zebu Azaouak	0.940	0.876	0.846	0.906	0.927	0.930	0.903	0.825	0.888	0.902	0.737	0.893	0.846	0.925	0.694			0.622	0.761	-0.034
17. Zebu Peul	0.895	0.846	0.805	0.865	0.896	0.887	0.868	0.799	0.838	0.864	0.725	0.877	0.819	0.899	0.695	0.917		0.469	0.805	0.035

Supplementary Figure S1. Network tree illustrating the genetic variability found with the haplotypes constructed using two IMMs and six Y-microsatellite loci typed on 248 zebu sire DNA samples. Network tree was constructed using the program Network 4.5 via computing a reduced median tree on which a median joining network method was applied. The 'frequency >1' option useful to discard singly occurring Y types was not applied to show the whole reticulations obtained. The same weights were assigned to each polymorphism. The three identified haplotypic families ($Y3_A$, $Y3_B$ and $Y3_C$) are, respectively, in orange, blue and green. Size of the circles corresponds with sample size. Missing haplotypes are identified by black small circles.

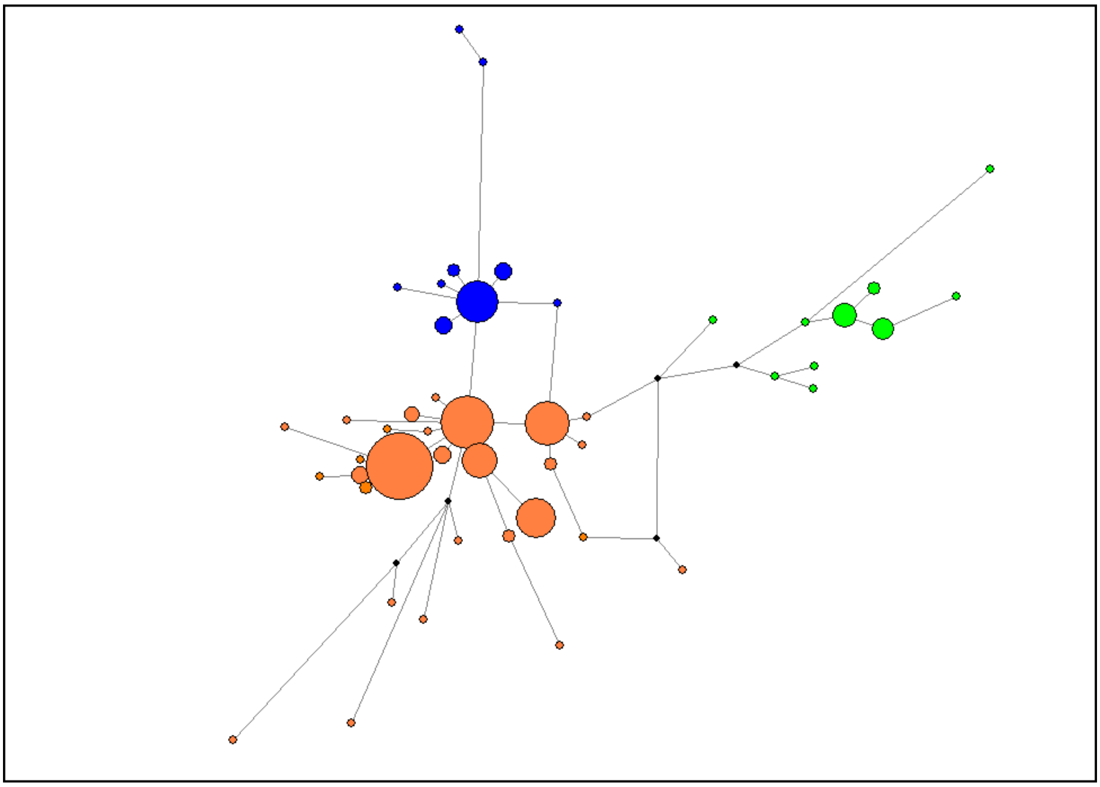

Supplementary Figure S2. Synthetic maps illustrating geographic variation of the first (Plot A) and second (Plot B) principal components identified using PCA. The first component of variation represents the contrast between the haplotypic family ($Y3_A$), predominant in the cattle breeds sampled in the Indian subcontinent and East Africa (the lighter colour the higher the frequency of $Y3_A$ haplotypes) and those areas in which the haplotypic family $Y3_C$ is present (darker colour). The second component represents the contrast between the haplotypic family $Y3_C$ (darker colour) and West Africa in which the haplotypic family $Y3_B$ is present (lighter colour). Sampling locations are also given.

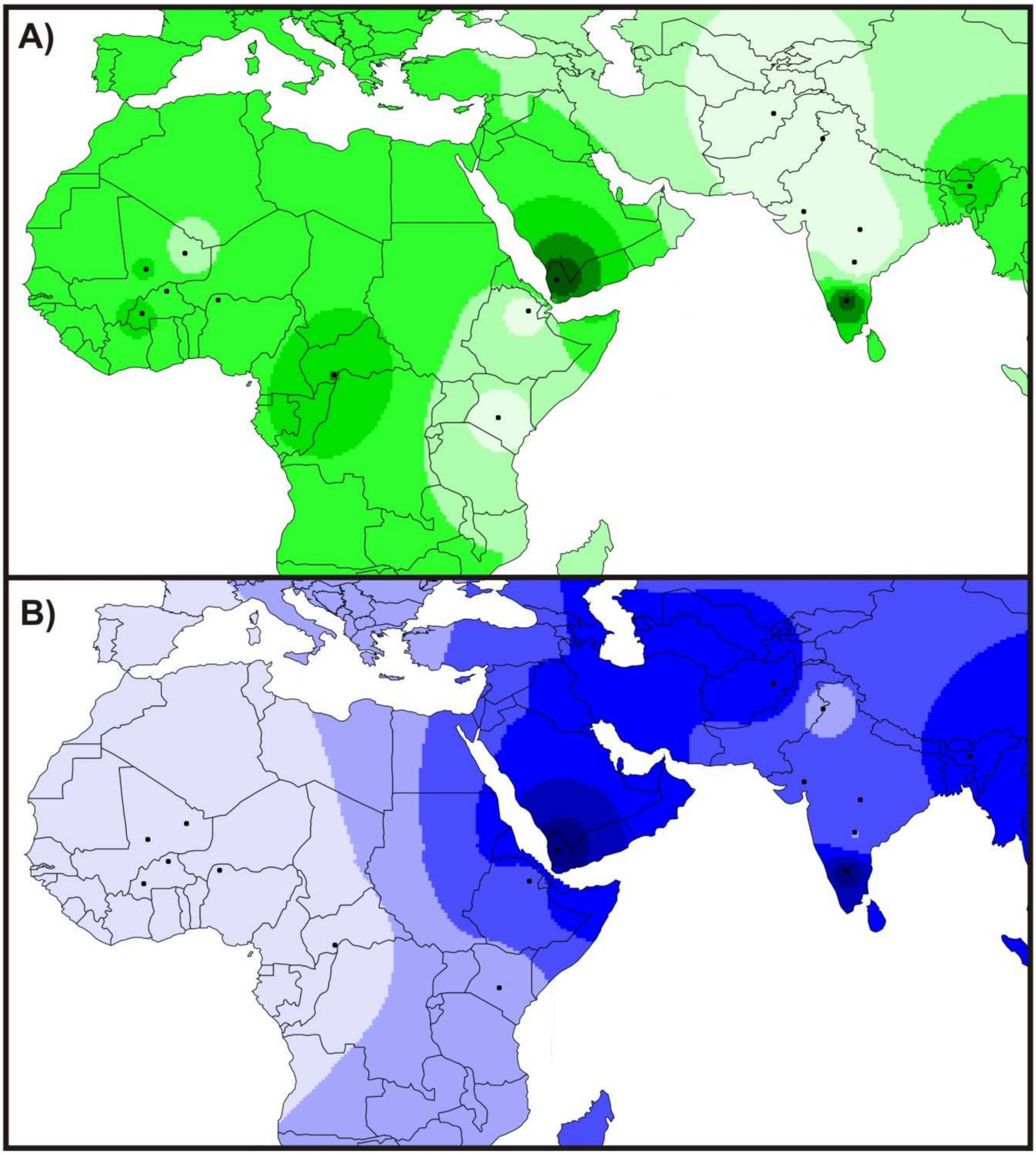

Supplementary Figure S3. Results of the ABC model choice misclassification. (A): ABC model choice misclassification. Number of correct (dark grey) and incorrect (light grey) allocations made by the ABC model choice using PODS (pseudo-observed data sets) from the three competing models. (B): Model 2 misclassifications. Number of PODS from Model 1 and Model 3 incorrectly allocated either to other model (light grey) or specifically to Model 2 (dark grey) in the ABC model choice. (C) and (D): Posterior probabilities supporting Model 2 in ABC model choices in which this model was incorrectly selected as the best model. Red lines indicate the posterior probability of Model 2 given the zebu empirical data. (E): Empirical calibration ABC posterior probabilities. Comparison of the ABC posterior probabilities (x-axis) and empirical probabilities obtained from PODS of the three models (y-axis) of Model 2. Red dashed line indicates the expected relationship with no bias.

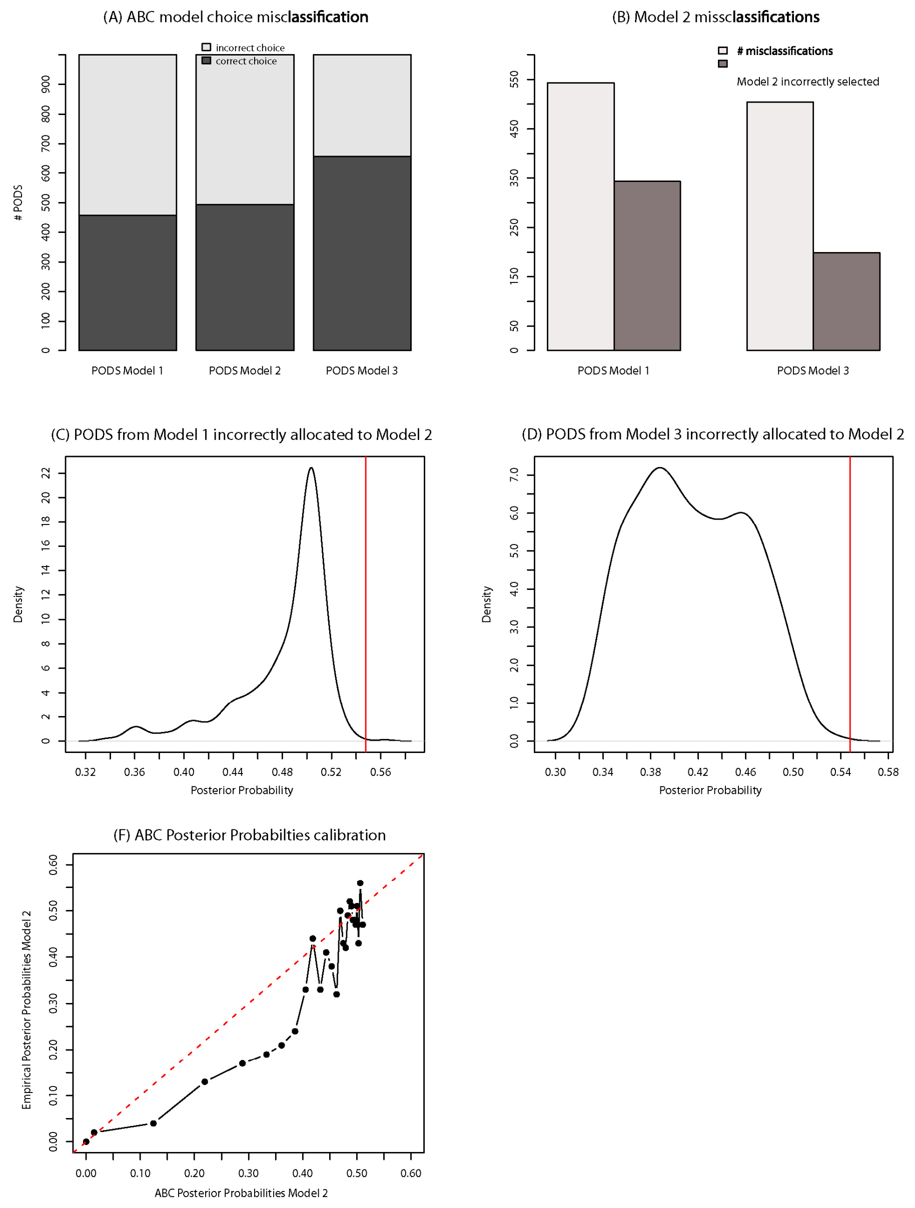

Supplement: Supplementary file 1 — Supplementary Information [file 41598_2018_36444_MOESM1_ESM.pdf]
